# Polyphenol-Enriched Fraction from Chestnut Shells as a Source of Bioactive Compounds for Friedreich Ataxia

**DOI:** 10.3390/molecules31010070

**Published:** 2025-12-24

**Authors:** Giuseppe Squillaci, Grazia M. Cotticelli, Virginia Carbone, Avery O. Westfall, Robert B. Wilson, Alessandra Morana

**Affiliations:** 1 Institute of Biochemistry and Cell Biology, National Research Council of Italy (CNR), Via Pietro Castellino 111, 80131 Naples, Italy; giuseppe.squillaci@cnr.it; 2 Department of Pathology and Laboratory Medicine, Children’s Hospital of Philadelphia, 3501 Civic Center Blvd., Philadelphia, PA 19104, USA; cotticellm@chop.edu (G.M.C.); westfalla1@chop.edu (A.O.W.); wilsonr@pennmedicine.upenn.edu (R.B.W.); 3 Institute of Food Sciences, National Research Council of Italy (CNR), Via Roma 64, 83100 Avellino, Italy; virginia.carbone@cnr.it; 4 Department of Pathology and Laboratory Medicine, Perelman School of Medicine, University of Pennsylvania, 3400 Spruce St., Philadelphia, PA 19104, USA; 5 Research Institute on Terrestrial Ecosystems, National Research Council of Italy (CNR), via Pietro Castellino 111, 80131 Naples, Italy

**Keywords:** chestnut shells, ferroptosis, Friedreich Ataxia, neurodegeneration, *p*-hydroxybenzoic acid, polyphenol-rich extracts, protocatechuic acid

## Abstract

We explored the ability of the low molecular weight, polyphenol-rich fractions obtained from chestnut shells to inhibit ferroptosis in Friedreich Ataxia (FRDA), an inherited neuro- and cardio-degenerative disease. We prepared an aqueous extract by an eco-sustainable method and obtained a polyphenol-rich fraction (fraction D) of molecular weight less than 1.0 kDa after molecular size fractionation. The total phenols were 173.28 ± 4.97 μg gallic acid equivalents/mg fraction, and analysis by UHPLC-ITMS^n^ and RP-HPLC-UV revealed thirteen phenolic compounds with gallic acid and protocatechuic acid (PCA) as the most abundant (26.29 ± 2.19 and 4.93 ± 0.19 μg/mg fraction, respectively). Using a cellular assay based on patient-derived FRDA fibroblasts, we observed that chestnut shell dry extract at 20 µg/mL increased the survival of cells stressed with the ferroptosis inducer erastin from 8% to 45% and that this activity was dose-dependent. Fraction D at 20 µg/mL showed similar strong activity, increasing cell survival from 0.5% to 14% and decreasing lipid peroxidation by 42%. PCA, the most efficacious compound, doubled cell survival and decreased lipid peroxidation by 20%. Moreover, PCA increased the survival of cells in which frataxin was knocked down 1.5-fold and decreased ALOX12 expression. Our data suggest that PCA could be a promising molecule to explore FRDA pathophysiology.

## 1. Introduction

The ever-increasing interest in the application of molecules of natural origin in many fields, including the therapeutic sector, has inspired the search for new natural sources to extract these beneficial compounds. Furthermore, investigations into new applications of already known natural molecules are also a constantly growing field. The study of ferroptosis inhibition aligns with this trend by attempting to identify natural molecules capable of intervening against this mechanism of programmed cell death.

Friedreich Ataxia (FRDA), the most common form of inherited ataxia, is a neurodegenerative disease that presents generally in late childhood as difficulties with balance and coordination [1]. Over the years, the progressive degeneration of large sensory neurons in the dorsal root ganglia causes ataxia, areflexia, sensory loss, and in some cases vision and hearing impairment. While all patients experience neurological symptoms, many of them present important co-morbidities such as diabetes and hypertrophic cardiomyopathy, which is the leading cause of premature death [1,2].

FRDA is caused, in the large majority of patients, by (GAA) repeat expansions in the first intron of the frataxin (*FXN*) gene on both alleles. In a small percentage of patients, the repeat expansion is present on one allele, and a point mutation or small deletion is found on the other allele [3]. The net effect of these mutations is the decreased expression of the functional frataxin protein. Frataxin protein localizes to mitochondria where it is involved in the biosynthesis of iron sulfur clusters, which are prosthetic groups present in many proteins. Thus, decreased frataxin levels affect a variety of biological processes. The most studied is mitochondrial dysfunction characterized by decreased ATP production, mitochondrial iron accumulation, and increased oxidative stress due to the overproduction of Reactive Oxygen Species (ROS) [3]. Oxidative stress has been long considered a hallmark of FRDA [4] but, more recently, lipid peroxidation and ferroptosis have been shown to be involved in the pathogenesis of the disease [5,6].

**Figure 1:**
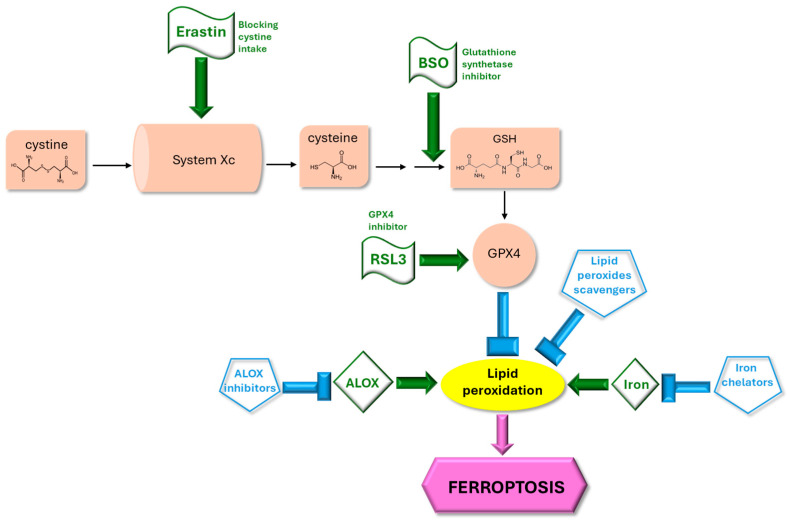
Modulation of ferroptosis schematic. Accumulation of lipid hydroperoxides drives cells to death. The glutathione-dependent enzyme GPX4 reduces toxic lipid hydroperoxides to lipid alcohols. Erastin, BSO, and RSL3 impair GPX4 activity, sensitizing cells to ferroptosis. Iron overload and possibly ALOX activity also sensitize cells to ferroptosis. Iron chelators, lipid peroxide scavengers, and possibly ALOX inhibitors render cells ferroptosis-resistant. Blue arrows, inhibition of ferroptosis; green arrows, triggers of ferroptosis.

Ferroptosis is a type of caspase-independent programmed cell death, distinct from apoptosis [7]. It can be triggered by iron overload, which results in a critical accumulation of lipid hydroperoxides (LOOH) that drive cells to death [8]. Lipoxygenase (ALOX) activity may also contribute to the increase in lipid hydroperoxides [9]. The endogenous defense mechanism against unintended ferroptosis relies on the activity of glutathione peroxidase 4 (GPX4), the enzyme that reduces lipid hydroperoxides into the corresponding lipid alcohols [8]. Direct inhibition of GPX4 by small molecules such as RSL3 [10], or glutathione synthesis inhibition by L-buthionine (S,R)-sulfoximine (BSO), are known mechanisms to sensitize cells to ferroptosis [6]. Blocking the membrane system Xc antiporter using small molecules such as erastin is another mechanism by which ferroptosis can be induced [10]. System Xc is a membrane antiporter that exchanges glutamate with cystine; the cystine is eventually converted to cysteine, which is required for glutathione synthesis. Conversely, iron chelators such as deferiprone [3], membranes enrichment with oxidation-resistant phospholipids [5], ROS scavengers, and ALOX inhibitors may interfere with the initiation and/or the progression of ferroptosis [3,9] (Figure 1).

Antioxidant therapy has been long considered a therapeutic approach for FRDA. Small molecules that activate genes downstream of the Antioxidant Response Element (ARE) have been tested in (Phase I/II) clinical trials [11]. In 2023, the synthetic molecule omaveloxolone (OMAV), an activator of the Nuclear factor Erythroid 2-related Factor 2 (NRF2) pathway, was the first drug to be approved for FRDA treatment [12]. NRF2—otherwise known as NFE2L2—is a transcription factor that increases the transcription of ARE-containing genes and is also a negative regulator of ferroptosis [13].

Although the approval of OMAV is a milestone for the field, the search for disease-modifying molecules is still very active. As evidenced by the recent literature, polyphenols are among the candidates being studied due to their recognized antioxidant power. Many flavonoids, a class of polyphenols, have been identified as natural modulators of ferroptosis [14]. Other types of polyphenols which are structurally very different from each other, such as the stilbene, resveratrol, the lignan nordihydroguaiaretic acid, and curcumin, chemically known as diferuloylmethane, possess anti-ferroptotic activity [11,15].

Polyphenols represent a large class of compounds with well-known valuable properties, ranging from antioxidant to anti-inflammatory, antitumor, and neuroprotective effects [16].

The exploitation of plant sources to produce polyphenol-rich extracts is nowadays mainly directed towards plant waste. In a circular economy model, materials traditionally considered waste are recommended as a novel resource, in agreement with the Sustainable Development Goal 12 (responsible consumption and production) of Agenda 2030, established in 2015 by the UN, target 12.5: “substantially reduce waste generation through prevention, reduction, recycling and reuse” [17,18].

Chestnut trees (*Castanea* sp.) belong to the *Fagaceae* family and are considered a substantial plant in forestry and the agricultural economy. Wood and bark are rich in tannins and chestnut leaves abound in bioactive components [19,20]. In fact, chestnut leaf extracts are endowed with strong anti-inflammatory power due to the presence of polar lipids [21]. Burs and shells are a reservoir of phenolic compounds [22,23,24]. The chestnut fruit has been consumed since ancient times, when it was considered the “poor man’s food”. Nowadays, chestnuts are consumed roasted or boiled or processed into various products such as *marrons glacés* and cakes, which are particularly appreciated by individuals following a gluten-free diet.

Over the last decades, the chestnut industry has significantly grown and in 2023, 313,078 tons of chestnuts were produced in Southern Europe with Spain as the leading producer followed by Italy which provided about 23% of European chestnut production (FAOSTAT, Food and Agriculture Organization of the United States: http://www.fao.org/faostat/en/#data, accessed on 27 October 2025). The chestnut peeling process, required for the isolation of the fruit, produces a solid waste represented by the chestnut shells (CS), which account for 10 to 15% of the whole chestnut weight. Consequently, the chestnut industry produces tons of CS, which are burned as fuel in the industrial plants to avoid disposal issues. However, it is now recognized that CS contains bioactive molecules that can be put to use rather than being burned.

The aim of the present paper was to identify new active polyphenols that could be protective in cellular models of FRDA. We focused our search on CS as the source of bioactive molecules. We adopted an eco-friendly and easily scalable extraction procedure for the preparation of a polyphenol-rich extract which was then subjected to molecular weight fractionation and purification. We tested the anti-ferroptotic activity of the polyphenolic extract and the purified fractions and identified protocatechuic acid (PCA) as the most efficacious component. Moreover, PCA was able to rescue human fibroblasts from the loss of viability induced by frataxin knock-down and to decrease the expression of ALOX in patient-derived cells. Taken together, our data identify PCA as a novel “molecule of interest” for FRDA treatment.

## 2. Results and Discussion

### 2.1. Production of Chestnut Shell Extract and Molecular Weight Fractionation

Chestnut shells were treated with boiling water to extract the phenolic compounds. Conventional solid–liquid extraction was selected among the previously evaluated extraction techniques because it provides a greater quantity of bioactive molecules [23]. Furthermore, this methodology is simple to perform and can be easily applied to large quantities of shells. Under our experimental conditions, the extraction yield was 10.0 ± 0.3% and the aqueous extract, which was freeze-dried (chestnut shells dry extract, CSDE) before proceeding to the next steps, contained 512 ± 7.8 μg gallic acid equivalents (GAE)/mg CSDE of total phenolic compounds.

It has been established previously that chestnut shells are a good source of tannins. Our data agreed with what has been reported by other authors as the total tannin content in CSDE accounted for 66% (338 ± 2.5 μg GAE/mg CSDE) of the total phenols detected [25,26]. Tannins are widespread in the plant kingdom and are classified as hydrolysable tannins and condensed tannins, with molecular weights ranging from 1.0 to 3.0 kDa and from 1.0 to over 20.0 kDa, respectively [27]. Given these ranges, high and low molecular weight active compounds in CSDE were separated by ultrafiltration membranes of different cut-offs. The procedure resulted in four fractions: specifically, from 1 g of CSDE, a dark brown sample (fraction A, 670.00 ± 8.56 mg) containing compounds with molecular weight higher than 30.0 kDa, two light brown samples with compounds of molecular weight higher than 10.0 kDa (fraction B, 4.18 ± 0.28 mg) and 1.0 kDa (fraction C, 6.43 ± 0.39 mg), and a pale-yellow sample (fraction D, 171.92 ± 2.37 mg) containing molecules of molecular weight less than 1.0 kDa were obtained. The phenolic content and tannin content of CSDE and fractions A and D are shown in Table 1.

The total recovery was 85%, with fractions A and D comprising 67% and 17% of CSDE, respectively. The 15% loss observed was likely due to membrane fouling phenomena caused by the repeated concentration and dilution steps during diafiltration. Fraction A contained 444.47 ± 11.16 μg GAE/mg fraction, the highest total phenolic content (TPC). The TPC in the whole fraction was 297.79 ± 8.94 mg GAE, which corresponded to 58.16% of the total phenols measured in CSDE. This fraction also had a notable content of total tannins, 384.70 ± 7.38 μg GAE/mg fraction, which was 50.34% of CSDE total phenols. The TPC of fraction D was 173.28 ± 4.97 μg GAE/mg fraction, while the quantity in the whole fraction was 29.79 ± 2.06 mg GAE and represented 5.82% of the total phenols of the extract. The yield of fractions B and C was so low that the determination of polyphenols and tannins was not carried out.

### 2.2. Characterization of Fraction D and Purification

**Figure 2:**
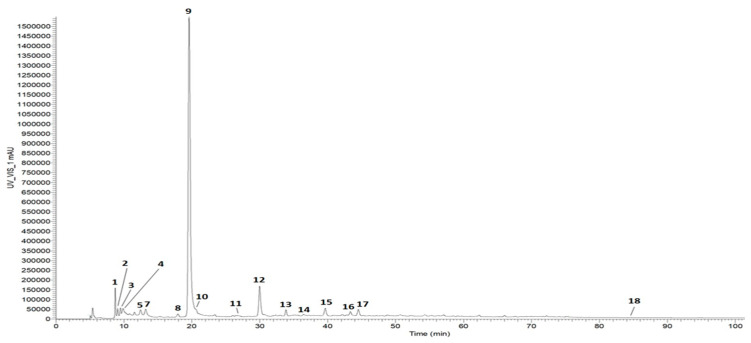
HPLC-UV chromatogram of phenolic compounds detected in fraction D recorded at 280 nm. For chromatographic conditions, see Methods section. For peak assignments, see Table 2.

Fraction D, containing compounds with molecular weights less than 1.0 kDa, was chosen to further investigate the anti-ferroptotic activity detected in CSDE. To ascertain the molecules responsible for the observed activity, the identification of the phenolic compounds in the fraction and their quantification were carried out by ultra-high performance liquid chromatography ion trap mass spectrometry (UHPLC-ITMS^n^) and RP-HPLC-UV analyses. The HPLC-UV chromatogram of the phenolic compounds is shown in Figure 2. The list of identified compounds is reported in Table 2.

Our analysis led to the detection and tentative identification of seventeen compounds based on the interpretation of their fragmentation patterns obtained from mass spectra (MS^n^ experiments); in addition, one compound was detected but not identified. Thirteen were phenolic compounds, whereas four belong to different classes, namely three organic acids—malic acid (Peak 1), tartaric acid (Peak 2), and quinic acid (Peak 5)—and the triterpenoid bartogenic acid (Peak 18). The three major peaks, gallic acid (GA) (Peak 9), protocatechuic acid (PCA) (Peak 12), and *p*-hydroxybenzoic acid (PHBA) (Peak 15) were selected for individual testing as anti-ferroptotic agents, in addition to the whole fraction. Quantification revealed concentrations of 26.29 ± 2.19, 4.93 ± 0.19, and 5.67 ± 0.75 μg/mg fraction, respectively. Among the remaining identified compounds, the majority were hydrolysable tannins.

**Figure 3:**
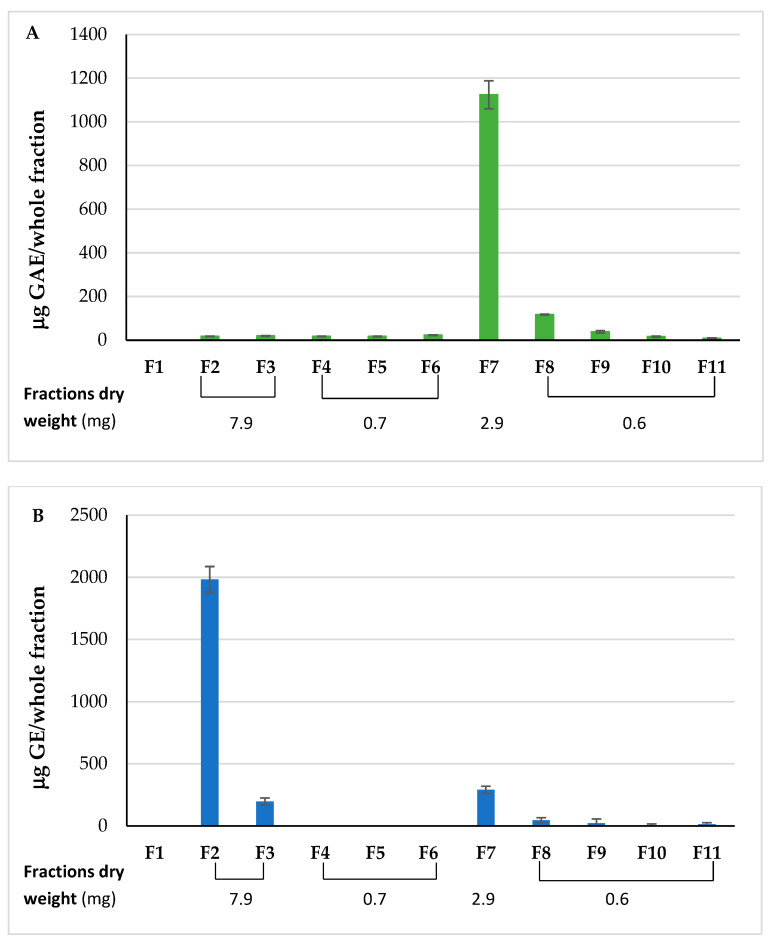
Total phenolic content (**A**) and total reducing sugars (**B**) detected in fractions from C18 purification of fraction D. GAE: Gallic Acid Equivalents; GE: Glucose Equivalents; F: Fraction. All determinations were performed in triplicate and results are expressed as mean ± SD.

To confirm that the anti-ferroptotic activity was due to the phenolic compounds, fraction D was subjected to a purification step by a C18 preparative column. Eleven fractions (F) were obtained: F1 (column packing water), F2 (unretained compounds), F3–6 (eluted with water, expected to contain the most polar compounds), and F7–11 (eluted with methanol, expected to contain the phenolic compounds). All fractions were analyzed to measure the total phenolic and sugar contents, and the results are shown in Figure 3.

Fraction 7 contained the largest quantity of phenolic compounds with 1124.27 ± 64.40 μg GAE in the whole fraction (Figure 3A). A very low quantity of phenolic compounds was detected in fractions 8–11. Sugars were mainly recovered in fraction 2, with 1982.33 ± 103.90 μg Glucose Equivalents (GE)/whole fraction, followed by fraction 3 (197.00 ± 27.87 μg GE/whole fraction) (Figure 3B). No further sugars were measured in the remaining fractions eluted with water, but they were again detected in the fractions eluted with methanol and containing the phenolic compounds. The positive results of the total-sugars-detection assay in fractions 7–11 could be due to the presence of hydrolysable tannins, which include an ester bond between a phenolic molecule and a sugar (Table 2). However, the total sugars recovered in these fractions accounted for 14.7% of the total sugars measured.

The purification by the C18 column separated the phenolic compounds from the sugars and concentrated the polyphenols in the first fraction eluted with methanol (fraction 7), which contained 80.5% of the total phenols recovered.

**Figure 4:**
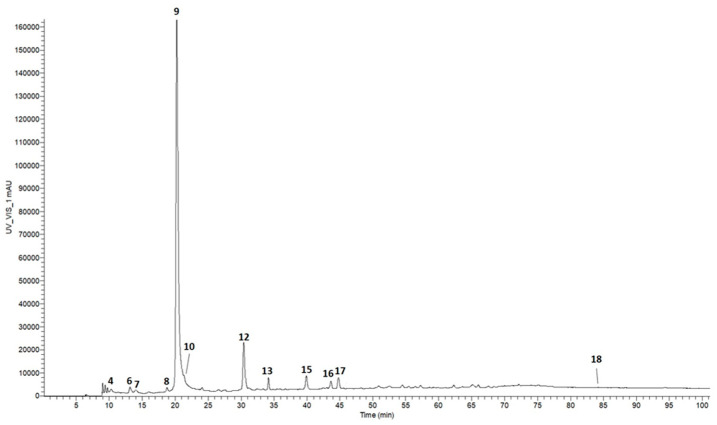
HPLC-UV chromatogram of phenolic compounds detected in fraction 7, obtained from the subsequent fractionation of fraction D, recorded at 280 nm. For chromatographic conditions, see Methods section. For peak assignments, see Table 2.

Qualitative and quantitative analyses of the phenolic molecules present in fraction 7 were performed by UHPLC-ITMS^n^ and RP-HPLC-DAD. The HPLC-UV chromatogram of the detected compounds is shown in Figure 4. The identified molecules are listed in Table 2. In particular, the qualitative composition of fraction 7 was similar to fraction D and contained GA (Peak 9), PCA (Peak 12), and PHBA (Peak 15) as the main phenolic molecules. They accounted for 214.07 ± 12.15, 44.84 ± 2.19, and 8.00 ± 0.99 μg/mg fraction, respectively, with GA and PCA concentrated more than 8- and 10-fold, respectively, compared to fraction D. Organic acids were likely eluted in the water fractions due to their polar nature.

After analyses, the following fractions were pooled: fractions 2–3, fractions 4–6, and fractions 8–11. All pooled fractions were dried and weighed (Figure 3), and a recovery of 79.11% by weight was obtained. Fractions 2–3 and 7 were selected for further testing.

### 2.3. CSDE Protects Primary FRDA Fibroblasts from Ferroptosis

**Figure 5:**
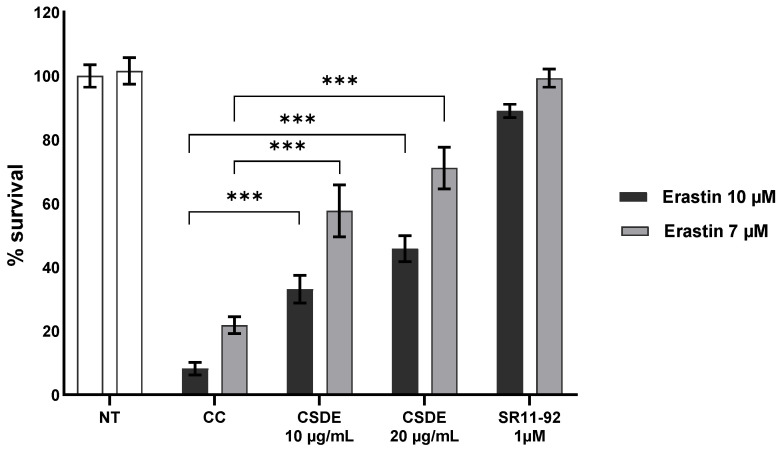
CSDE anti-ferroptotic activity. Primary, patient-derived FRDA fibroblasts Coriell 3816 were treated with the ferroptosis inducer erastin at 7 µM or 10 µM for 24 h, which resulted in significant cell death. Addition of CSDE at 20 µg/mL or 10 µg/mL 2 h after erastin treatment increased cell survival. Survival is calculated relative to cells not treated with erastin (NT); 1 µM SR11-92 was used as positive control for the assay and the statistical significance of the results with SR11-92 are not shown. CC = carrier control (water); CSDE = chestnut shells dry extract; *** = *p* < 0.005 by two-way ANOVA with Bonferroni post hoc test. The experiment shows the average and SD of five independent technical replicates and is representative of three independent experiments.

Primary, FRDA patient-derived fibroblasts have long been known to be sensitive to oxidative stress compared to apparently healthy, primary fibroblasts when treated with hydrogen peroxide [28] or BSO [29]. More recently, we showed that cells derived from two different murine models of FRDA, as well as human primary FRDA fibroblasts, are more sensitive to the ferroptosis inducer erastin [30] and we developed a microplate cellular assay to test compounds of possible relevance for FRDA treatment [31]. The assay is fairly specific for ferroptosis inhibitors as we have previously shown that apoptotic inhibitors are not efficacious [30], although we did not rule out every other type of programmed cell death. To compensate for the inevitable variability that comes from using primary cells and rapidly degrading reagents, we run multiple controls in each assay: cells not treated with a ferroptosis inducer, cells treated with the ferroptosis inducer to establish the efficacy of the treatment, and a positive control—i.e., cells in which the ferroptosis inducer treatment is followed by the addition of SR11-92, a classical ferroptosis inhibitor. The compounds to be tested are added after the addition of the ferroptosis inducer—mimicking what would happen in a real therapeutic intervention, in which a certain degree of damage has already occurred—and they are evaluated compared to controls. As Figure 5 shows, adding erastin to human primary FRDA fibroblasts at 7 µM or 10 µM for 24 h results in a dose-dependent loss of cell viability, with only 22% and 8% of cells surviving, respectively. Treatment of cells with 1 µM SR11-92 protected cells treated with 7 µM erastin almost completely (99%) and nearly as well (90%) when the cells were treated with 10 µM erastin.

**Figure 6:**
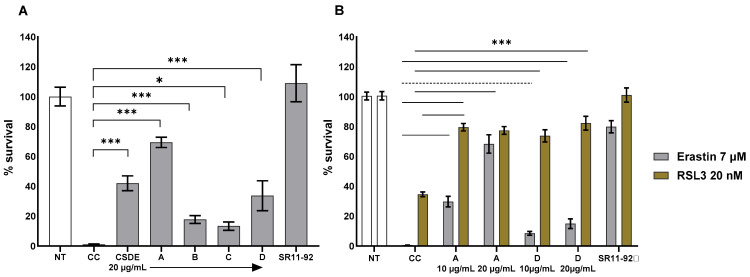
Comparative anti-ferroptotic activity in CSDE fractions. (**A**) Primary, patient-derived FRDA fibroblasts Coriell 3816 were treated with the ferroptosis inducer erastin at 7 µM for 24 h. Addition of CSDE and fractions A to D at 20 µg/mL 2 h after erastin treatment increased cell survival. (**B**) Primary, patient-derived FRDA fibroblasts Coriell 3816 were treated with 7 µM erastin or 20 nM RSL3 for 24 h. Treatment with fraction A and D at 20 µg/mL and 10 µg/mL increased cell survival. Survival is calculated relative to cells not treated with erastin or RSL3 (NT); SR11-92 1 µM was used as positive control for both assays and the statistical significance of the data with SR11-92 is not shown. CC = carrier control; *** = *p* < 0.005; dotted line = *p* < 0.01; * = *p* < 0.05 by one-way (**A**) or two-way ANOVA (**B**) with Bonferroni *post hoc* test. The experiment shows the average and SD of five (**A**) or four (**B**) technical replicates and it is representative of at least two independent experiments.

CSDE increased cell survival in a dose-dependent manner from 22% to 57% and 71% when used at concentrations of 10 µg/mL and 20 µg/mL, respectively, after treatment with 7 µM erastin. Similarly, when cells were treated with 10 µM erastin, CSDE increased survival from 8% to 33% and 45%, when used at concentrations of 10 µg/mL and 20 µg/mL, respectively. Following CSDE molecular fractionation, we tested the fractions A–D using the same assay. All four fractions were active in the assay as they all increased the survival of cells treated with erastin in a statistically significant way (Figure 6A).

Fractions B and C showed a similar activity as they increased the survival of cells treated with 7 µM erastin from 1% to 18% and 13%, respectively. Fraction D, containing low molecular weight polyphenols, showed a slightly lower activity than the total extract as survival increased to 34% compared to 41% using CSDE. Surprisingly, fraction A was not only active in the assay, but it increased survival to 69%. To further confirm these results, we tested whether fractions A and D were able to increase cell survival when RSL3 was used. In contrast with erastin, RSL3 is an inhibitor of GPX4, the enzyme responsible for lipid hydroperoxides reduction, and its activity is intracellular (Figure 1). We found both fractions A and D (Figure 6B) to be efficacious. Although we were interested primarily in the low molecular weight polyphenolic fraction, we were intrigued by these results, as a high molecular weight fraction, rich in tannins, was not expected to contain compounds that could cross the plasma membrane. In the erastin assay, which resulted in a 99% loss of viability, fraction A was able to increase survival to 68% when used at 20 µg/mL, more than 4-fold higher than fraction D did at the same concentrations. In the RSL3 assay, there was a higher survival at baseline and the loss of viability that followed the treatment was 66%. Fraction A and fraction D showed similar activity, improving survival to 73% and 82% when used at 20 µg/mL, respectively. We concluded that both fractions A and D can have anti-ferroptotic activity. It should be noted that in the RSL3 assay there is not a clear dose-dependency, as observed in the assay with erastin, perhaps due to the different kinetics by which the cells die in the two assays. The possible mechanism of action of fraction A is puzzling: one possible explanation for the activity could be that tannins contained in fraction A might release small amounts of catechin, which can then cross the plasma membrane. In fact, we found catechin efficacious in the erastin assay but only at concentrations between 100 µM (29 µg/mL) and 1 mM. Another possibility is that fraction A may decrease erastin binding to its receptor, System Xc (Figure 1). One or more fraction A components might absorb erastin. Alternatively, binding of one of the components of fraction A to an allosteric site of System Xc might interfere with erastin binding but still allow cystine import (Figure 1). However, the efficacy of fraction A in the assay with RSL3 ruled out these alternatives as the efficacy of fraction A is not erastin-dependent. Finally, one or more components of fraction A might chelate iron, decreasing the bioavailable iron in the medium and thereby decreasing cellular iron uptake. Consistent with this hypothesis, ferroptosis is an iron-dependent process (Figure 1) and iron chelators have been used as anti-ferroptotic agents [3]. We plan to investigate this activity in a future study, and we did not pursue it further here.

### 2.4. Polyphenol-Rich Fraction D Rescues Lipid Peroxidation Induced by RSL3 Treatment

**Figure 7:**
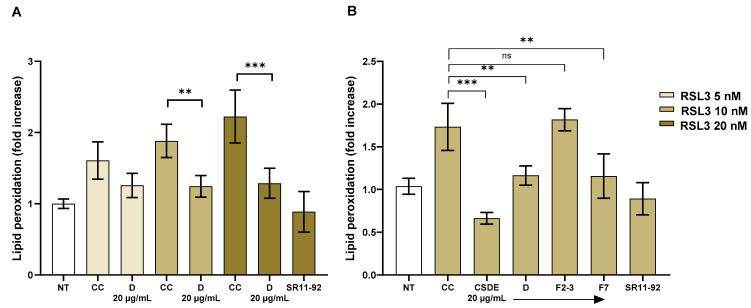
Polyphenol-rich fraction decreases lipid peroxidation. (**A**) Primary, patient-derived FRDA fibroblasts Coriell 3816 were treated with increasing doses of RSL3 for 24 h in the presence of carrier control or fraction D at 20 µg/mL. The following day, C-11 Bodipy™ dye was added at 10 µM for 30 min. Fluorescence was measured on a plate reader using 485/520 (ex/em) and 485/590 (ex/em) filters. An increase in the 520/590 ratio is indicative of lipid peroxidation. (**B**) FRDA fibroblasts Coriell 3816 were incubated overnight with 10 nM RSL3 and treated with CSDE, fraction D, F2–3, or F7 all at concentrations of 20 µg/mL. Lipid peroxidation was measured as described above. Data are represented as fold increase in lipid peroxidation relative to cells not treated with RSL3 (NT). SR11-92 1 µM was used as a positive control for both assays and the statistical significance of the SR11-92 data is not shown. CC = carrier control; CSDE = chestnut shell dry extract; *** = *p* < 0.005; ** = *p* < 0.01 by one-way ANOVA with Bonferroni *post hoc* test. The experiment shows the average and SD of four independent technical replicates, and it is representative of at least two independent experiments.

To measure lipid peroxidation, we used Bodipy C11™ dye, which undergoes a fluorescence shift following oxidation. The ratio of the fluorescence emission of the [oxidized] form to the fluorescent emission of the [reduced] form is a direct measure of lipid peroxidation in the cell. We treated human primary FRDA fibroblasts with increasing concentrations of RSL3 and used Bodipy C11™ to measure the ratio of oxidized/reduced lipids (Figure 7A). Lipid peroxidation was increased 2.2-fold when cells were treated with RSL3 at 20 nM but the addition of fraction D at 20 µg/mL lowered this increase to 1.29-fold (42% less). Similarly, in cells treated with RSL3 at 10 nM, fraction D lowered the increase in lipid peroxidation (34% less). When RSL3 was used at 5 nM, there was still a 1.51-fold increase in lipid peroxidation, but the activity of fraction D did not reach statistical significance.

At least one disaccharide, trehalose, has been shown to have anti-ferroptotic activity [32], whereas monosaccharides such as glucose may contribute to ferroptosis through promoting iron overload [33]. To confirm that the observed anti-ferroptotic effect was attributable to the polyphenolic components, fraction D was further separated into sugar-containing fractions (fractions 2–3) and a polyphenol-enriched fraction (fraction 7), which were tested independently. The results confirmed that the activity in fraction D was due to the polyphenolic fraction 7 (Figure 7B), which caused a 30% decrease in lipid peroxidation, to the same extent as fraction D did. The sugar-containing fractions 2–3 showed no activity. Of note, the total extract decreased lipid peroxidation to a level lower than untreated cells.

We conclude that fraction 7 recapitulates the activity of fraction D. It should also be noted that we used conditions in which lipid peroxidation increased with increasing concentrations of RSL3. However, fraction D decreased lipid peroxidation to a similar extent (42% and 34%) bringing the ratio to a similar value, 20% higher than basal. Unfortunately, this assay has a very limited dynamic range, as a few-fold increase in lipid peroxidation is sufficient to cause cell death in primary FRDA fibroblasts. We tried to decrease the concentration of fraction D and/or RSL3, but the data lost all statistical significance.

### 2.5. Protocatechuic Acid Increases Survival of FRDA Fibroblasts

**Figure 8:**
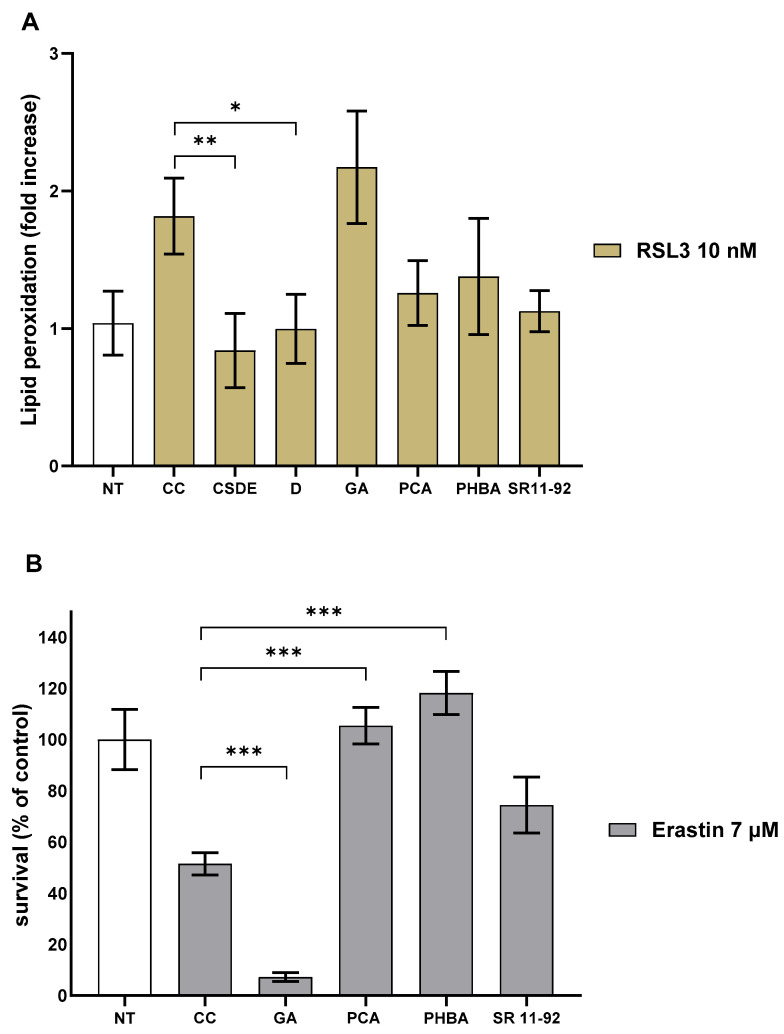
PCA decreases lipid peroxidation and increases survival. (**A**) Primary, patient-derived FRDA fibroblasts Coriell 3816 were treated with 10 nM RSL3 for 24 h in the presence of carrier control (CC), 2.5 µM GA, 0.5 µM PCA, or 0.82 µM PHBA. The following day, C-11 Bodipy™ dye was added at 10 µM for 30 min. Fluorescence was measured on a plate reader using 485/520 (ex/em) and 485/590 (ex/em) filters. An increase in the 520/590 ratio is indicative of lipid peroxidation. Data are represented as the fold increase in lipid peroxidation relative to cells not treated with RSL3 (NT). (**B**) Primary, patient-derived FRDA fibroblasts Coriell 3816 were treated with 7 µM erastin for 24 h. Addition of 0.5 µM PCA or 0.82 µM PHBA increased survival whereas 2.5 µM GA caused toxicity. SR 11-92 1 µM was used as a positive control for both assays and the statistical significance of the SR 11-92 data is not shown. CC = carrier control; CSDE = chestnut shell dry extract; *** = *p* < 0.005; ** = *p* < 0.01; * = *p* < 0.05 by one-way ANOVA with Bonferroni *post hoc* test. The data shown are the mean and the standard deviation calculated from three independent technical replicates, and they are representative of at least two independent experiments.

Following the identification of the most abundant compounds in the polyphenolic fraction 7, we tested commercially available GA, PCA, and PHBA in the lipid peroxidation assay and in the erastin assay. We used the compounds at the same concentration at which they were present in fraction D (20 µg/mL). We treated human primary FRDA fibroblasts with 1 nM RSL3 and found that 0.5 µM PCA decreased lipid peroxidation from 1.8-fold to 1.3-fold (18% less), a result almost identical to the entire fraction D (20 µg/mL) (Figure 8A). PHBA at 0.82 µM also decreased lipid peroxidation to 1.4-fold. In contrast, the addition of 2.5 µM GA resulted in an increase in lipid peroxidation to 2.2-fold (Figure 8A). However, when we used the less stringent two-sided Student *t* test, the decreased induced by PCA was the only one to become statistically significant (*p* = 0.034). Finally, we tested the active compounds in the erastin assay (Figure 8B).

We found that 0.5 µM PCA could increase the survival of cells treated with 7 µM erastin from 51% to 105% and 0.82 µM PHBA was also able to increase rescue from 51% to 118%. Consistent with the previous results, the addition of 2.5 µM GA resulted in cell toxicity, as survival went from 51% to 7%. Of note, treatment of human primary FRDA fibroblasts, in which ferroptosis was not induced, with fraction 7 (20 µg/mL), 0.5 µM PCA, 0.82 µM PHBA, and 2.5 µM GA showed that the compounds had no effect on cell growth and did not induce any cell toxicity. It should also be remarked that we only tested the compounds at one concentration, which may have not been the optimal one for each assay. There are numerous reports about GA in ferroptosis, and its activity seems to be context-dependent. While GA may have a protective effect in traumatic brain injury [34], in cancer cell lines like SHSY5Y or HeLa, GA can induce ferroptosis when used at a concentration of 300 µM [35]. Of note, in our system, when ferroptosis is induced, GA increases toxicity at a much lower concentration, confirming the sensitivity of FRDA cells to ferroptosis. Considering the toxicity of GA, it is impressive that fraction D and fraction 7, in which GA is highly represented, are beneficial to the cells. On the other hand, GA was not toxic when added to cells in which ferroptosis was not induced, supporting the hypothesis that toxicity was not due to the GA dose itself. One possible explanation is that the combined mixture of polyphenols might prevent the cells from reaching a point at which GA could amplify the damage, at least during the time frame of the assay. In retrospect, GA toxicity may also explain why fraction D was never able to decrease lipid peroxidation completely (Figure 7A). To date, there are no reports of PHBA activity in ferroptosis. PHBA administration has been shown to increase CoQ10 biosynthesis [36], which in turn can contribute to suppressing ferroptosis [37]. Idebenone, a CoQ10 analog, has also been tested in FRDA patients [11]. The short time frame of our assay (24 h) would suggest that the ROS scavenger activity of PHBA may play a role. Our data show that PHBA was more efficacious in increasing survival than in decreasing lipid peroxidation. More studies are needed to investigate the mechanism of action of PHBA in ferroptosis.

**Figure 9:**
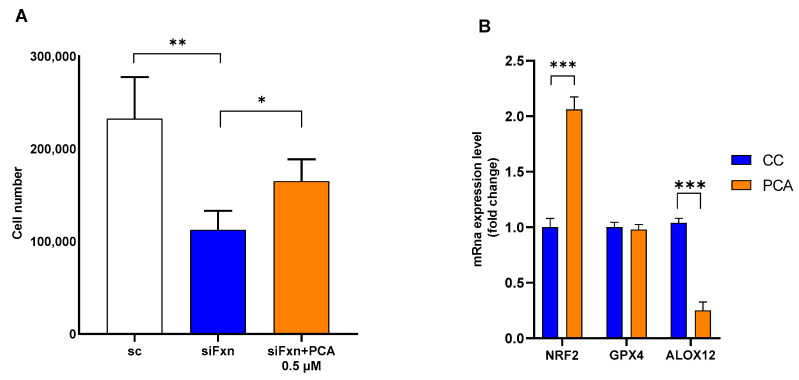
PCA treatment of cells with low frataxin expression. (**A**) Human, healthy, primary fibroblasts (Coriell 8400) were transfected with anti-Fxn siRNA (siFxn) or scrambled control (sc) on day 1. On days 2, 4, and 5, 0.5 µM PCA was added in fresh medium. Cells were counted on day 6. (**B**) Relative expression of *NRF2*, *GPX4*, and *ALOX12* in human, primary FRDA fibroblasts Coriell 3816 treated with 0.5 µM PCA for 24 h. Data were normalized using TATA binding protein (TBP) as reference gene. siFxn, anti-Fxn siRNA; sc = scrambled siRNA; *** = *p* < 0.005; * = *p* < 0.05; ** = *p* < 0.01 by two-sided *t* test (**A**) or by two-way ANOVA (**B**). The data show the average and the SD calculated from three independent replicates in technical quadruplicate (**A**) or in triplicate (**B**) and are representative of at least three independent experiments.

In our assays, the most efficacious compound was PCA, which consistently decreased lipid peroxidation and increased cell survival when erastin or RSL3 was used as the ferroptosis inducer. These data are also in agreement with two recent reports that described a protective effect of PCA in ferroptosis [38,39]. To further explore PCA efficacy in FRDA cellular models, we tested whether PCA could rescue cells in which death was induced by frataxin knock-down. In contrast with the previous assays, knocking-down frataxin causes a loss of cell viability over six days. Moreover, we previously showed that during this time, lipid peroxidation does not increase substantially, but sensitivity to RSL3 does [30]. Transfection of apparently healthy, human primary fibroblasts (Coriell 8400) with short interfering RNA against frataxin results in a loss of cell viability compared to cells transfected with a control scrambled RNA. The addition of PCA at 0.5 µM every 48 h starting on the first day post-transfection increased survival from 52% to 71% (Figure 9A). The frataxin protein level was at that point 7% (Figure S1).

A recent study demonstrated that the anti-ferroptotic activity of PCA in a hepatic steatosis model is dependent on the up-regulation of *NRF2* leading the authors to suggest that PCA is a “functional NRF2 activator” [38]. Moreover, the NRF2 pathway has been shown to be down-regulated in FRDA patients’ cells [40]. Taken together, these data support the hypothesis that NRF2 activation is down-regulated following frataxin knock-down and can be restored, at least partially, by PCA under conditions in which ferroptosis is not induced acutely. To test whether PCA transcriptionally modulates the NRF2 pathway and other enzymes involved in ferroptosis (Figure 1), we treated patient-derived fibroblasts (Coriell 3816) with 0.5 µM PCA for 24 h and found a two-fold up-regulation of *NRF2*, no difference in GPX4 expression, and, unexpectedly, a 75% decrease in *ALOX 12* expression, the lipoxygenase most expressed in skin-derived fibroblasts (Figure 9B). Because *ALOX12* expression is quite low in fibroblasts, we repeated the experiment several times (*n* = 5) and found that the average expression of *ALOX12* in treated cells was 44 (±20) % of cells treated with the carrier control. We repeated the experiment using a different patient line (FRDA Cell Repository 4497), with comparable results. OMAV, the approved treatment for FRDA, also decreased *ALOX12* expression (Figure S2). While thought-provoking, the full assessment of these data’s significance will require measurements of the ALOX12 protein level and especially enzymatic activity. These results are in agreement with what has been reported by La Rosa et al. [40], who found that the NRF2 pathway was up-regulated in patient cells treated with EPI-743 (vatiquinone), an ALOX inhibitor. The inhibition of lipoxygenase activity is considered a viable target in FRDA [3,11]. Furthermore, PCA was also reported as a quercetin metabolite bound to lipoxygenase [41]. At this time, we lack a convincing mechanistic explanation regarding how PCA might regulate *NRF2* up-regulation, and whether *ALOX12* down-regulation is dependent on, or independent of, *NRF2* up-regulation. It is possible that the antioxidant response regulated by *NRF2* downregulates lipoxygenase(s) expression through a yet uncharacterized mechanism. However, the data in La Rosa et al. [40] suggest that lipoxygenase inhibition precedes NRF2 pathway upregulation, which is counterintuitive. More data are required to gain a better understanding of the antioxidant response in FRDA.

In conclusion, we showed that PCA is protective in cellular models of FRDA. Our results are consistent with PCA being an ROS scavenger. The data support the hypothesis that *NRF2* activation could also contribute to the phenotypic benefit of PCA, especially when ferroptosis is not induced acutely. Moreover, we showed that PCA decreased the expression of *ALOX12*, which likely decreases ALOX12 activity. This is consistent with the beneficial effect we found of PCA on lipid peroxidation. It has been argued that all reported ALOX inhibitors are also ROS scavengers [9]; thus, distinguishing between the various mechanisms of action in play may prove challenging, especially in fibroblasts. Most interestingly, a role for *ALOX 12* has been described in cardiac fibrosis [42] and in type II diabetes [43], two of the most common and impactful co-morbidities of FRDA, the development of which are still poorly understood. Therefore, we plan to study PCA administration in more pathology-relevant systems.

## 3. Materials and Methods

### 3.1. Chemicals

Chemicals needed for the total phenolic content determination (Folin–Ciocalteu reagent, sodium carbonate), for the reducing-sugars assay (3,5-dinitrosalicylic acid, sodium hydroxide), cinchonine hemisulfate, glucose, gallic acid, protocatechuic acid, and *p*-hydroxybenzoic acid were purchased from Merck Life Science (Milano, Italy).

High performance liquid chromatography (HPLC) grade acetonitrile was obtained from Merck (Darmstadt, Germany). Acetic acid was purchased from Carlo Erba (Rodano, Milan, Italy). HPLC grade water (18.2 mΩ) was prepared using a Millipore Milli-Q purification system (Millipore Corp., Bedford, MA, USA).

Erastin (2-(1-(4-(2-(4-Chlorophenoxy)acetyl)-piperazin-1-yl)-ethyl)-3-(2-ethoxyphenyl)-3H-quinazolin-4-one), RSL3 ((1S,3R)-2-(2-Chloroacetyl)-2,3,4,9-tetrahydro-1-[4-(methoxycarbonyl)phenyl]-1H-pyrido [3,4-b]indole-3-carboxylic acid methyl ester), ethyl3-(benzylamino)-4-(cyclohexylamino) benzoate (SRS11-92), and OMAV (Omaveloxolone RTA 408- *N*-(2-Cyano-3,12-dioxo-28-noroleana-1,9(11)-dien-17-yl)-2,2-difluoropropanamide) were obtained from Selleckchem (Houston, TX, USA). Bodipy™ 581/591 C11 was obtained from Thermo Fisher (Waltham, MA, USA). All these compounds were solubilized in DMSO (Thermo Fisher, Waltham, MA, USA). Gallic acid, protocatechuic acid, and *p*-hydroxybenzoic acid were solubilized in methanol (UN 1230 Fisher Scientific, Norristown, PA, USA) and subsequently diluted in milliQ water.

### 3.2. Extract Preparation from Chestnut Shells

Chestnut shells from *Castanea sativa* Mill. were kindly provided by Terminio Frutta (San Michele di Serino, Avellino, Italy). The raw material was obtained after peeling the chestnut fruits through a drying process. This technique involves treating the chestnuts with hot air for several days and then peeling them using a peeling machine. The resulting dried shells were collected and used to prepare the extract as described below.

After drying the shells in oven at 55 °C, they were ground by a food homogenizer (Tefal, type 8557-54, Rumilly, France). The bioactive molecules were extracted as follows: 5% (*w*/*v*) chestnut shells were suspended in deionized water and boiled for 1 h under continuous stirring. After cooling, the suspension was centrifuged at 3220× *g* for 1 h at 4 °C (Eppendorf 5810R, Eppendorf s.r.l., Milano, Italy), and the supernatant was recovered. The spent residue was rinsed with the same amount of water lost during the boiling process and centrifuged as above. The second supernatant was added to the previous one to restore the original volume. The extract was freeze-dried in an Edwards Modulyo freeze-dryer (Edwards, Cinisello Balsamo, Milano, Italy), and the powder (CSDE) was stored at room temperature.

### 3.3. Fractionation of the Extract

A measurement of 1% (*w*/*v*) CSDE was solubilized in deionized water and centrifuged at 11,000 *g* for 45 min at 4 °C to remove small fragments of shells still present. The supernatant was transferred to Amicon ultrafiltration cell (EMD Millipore Corporation, Burlington, MA, USA) equipped with regenerated cellulose membranes of 30.0, 10.0, and 1.0 kDa Molecular Weight Cut-Off (EMD Millipore Corporation, Billerica, MA, USA). The diafiltration process started with 30.0 kDa membrane. When the retentate reached approximately 15 mL, the volume was restored to the initial value by adding deionized water and then subjected to a new diafiltration. This process was continued until no absorption at 280 nm was detected in the permeate. The final permeate was subjected to diafiltration with 10.0 kDa membrane following the above steps. Then, the permeate was diafiltered with 1.0 kDa membrane until no absorption at 280 nm was measured. Four samples were obtained at the end of the process: fraction A > 30.0 kDa, fraction B > 10.0 kDa, fraction C > 1.0 kDa, and fraction D < 1.0 kDa. All fractions were freeze-dried, weighed, and stored at room temperature.

### 3.4. Purification of Fraction D

Fraction D was solubilized in deionized water at a concentration of 15 mg/mL. One mL aliquots were loaded onto preparative Phenomenex Strata C18-E (55 µm, 70 Å) columns (Phenomenex, Torrance, CA, USA) previously washed with 8 column beds of methanol and equilibrated with 8 column beds of deionized water. While loading the sample, one fraction containing the column packing water and a second one, containing the unretained compounds, were collected (fractions 1 and 2, 0.5 mL each). The elution of the most polar compounds was carried out by flushing 8 column beds of deionized water (fractions 3–6, 1 mL each); then, the less polar molecules were eluted with 10 column beds of methanol (fractions 7–11, 1 mL each). Fraction 1 was discarded as it contained the column packing water; fractions 2 and 3, fractions 4–6, and fractions 8–11 were pooled. Fraction 7 was kept separate. Pools 2–3 and pools 4–6 were freeze-dried, whereas the remaining pools and fractions were dried under a stream of nitrogen. All fractions were weighed and stored at 4 °C until use. Before the biological assays, fractions A–D and the two D-subfractions 2–3 and 7 were prepared in milliQ water at concentration of 4 mg/mL.

### 3.5. Sample Characterization

All samples were analyzed for the estimation of the total phenolic content, whereas total tannins were measured in CSDE and fraction A. Fractions obtained after column separation were also analyzed for the determination of the total reducing sugars. All tests were conducted in triplicate and results were expressed as mean ± standard deviation (SD).

#### 3.5.1. Total Phenolic Content Assay

The total phenols were estimated by the Folin–Ciocalteu assay [44]. Aliquots of samples, appropriately diluted to 150 µL, were added to 750 µL of Folin–Ciocalteu reagent (1:10 in deionized water) and 600 µL of 7.5% (*w*/*v*) sodium carbonate. The assay solution was incubated at 25 °C in the dark for 2 h. The absorbance of the samples was measured at 765 nm with a blank containing 150 µL of deionized water (Thermo Scientific spectrophotometer, model Genesys 180, Rodano, Milan, Italy). The quantity of phenolic compounds was calculated by a calibration curve prepared with different amounts of gallic acid (1.5–10 μg) used as a standard. The results were expressed as μg GAE/mg CSDE or mg fraction.

#### 3.5.2. Total Tannin Content Assay

The estimation of the total tannins was performed as described in Squillaci et al. [22]. In brief, 0.8 mL of samples were mixed with the same volume of 0.5% (*w*/*v*) cinchonine hemisulfate. This solution was kept overnight at 4 °C to obtain better precipitation; then, it was centrifuged at 16,100× *g* for 5 min at 4 °C. The residue contained the tannin fraction while the non-tannin fraction was in the supernatant. The non-tannin content was measured by the Folin–Ciocalteu assay, and the total tannins were estimated by the difference between the total phenolic content and the non-tannin content. The results were expressed as μg GAE/mg CSDE or mg fraction.

#### 3.5.3. Reducing Sugars Assay

The reducing sugars were measured by the 3,5-dinitrosalicylic acid (DNS) reagent [45]. Aliquots of samples, appropriately diluted to 125 μL, were added to 100 μL of DNS in 2 M sodium hydroxide (1 g DNS in 10 mL of sodium hydroxide solution). The assay solution was boiled for 5 min, cooled on ice, and then mixed with 1 mL of deionized water. The absorbances of the samples were measured at 546 nm and compared to a blank prepared with 125 μL of deionized water. The quantity of reducing sugars was calculated using a calibration curve prepared with different amounts of glucose (0.3–1.25 μmoles) as a standard. The reducing sugars content was expressed as μg of Glucose Equivalents (GE) in the whole fractions.

#### 3.5.4. Ultra-High Performance Liquid Chromatography-Ultraviolet (UHPLC-UV) and UHPLC-Mass Spectrometry (UHPLC-MS^n^) Analyses

Fraction D, containing the molecules with molecular weights less than 1.0 kDa present in CSDE, and fraction 7, obtained from the subsequent fractionation of fraction D on a C18 preparative column, were analyzed by ultra-high performance liquid chromatography (UHPLC)-linear ion trap mass spectrometry on an LTQ XL ion-trap mass spectrometer coupled with a UHPLC Ultimate 3000 RS chromatographic system equipped with a Diode Array Detector and Xcalibur^®^ system manager data acquisition software v4.5 (Thermo Fisher Scientific, Waltham, MA, USA). Individual compounds were separated on Luna C18(2) column (250 × 4.6 mm, 5.0 μm, Phenomenex, Torrance, CA, USA) equipped with a SecurityGuard™ pre-column containing a C18 cartridge, at a flow rate of 500 μL/min; solvent A was 0.2% acetic acid, and solvent B was 0.1% acetic acid in acetonitrile and water (50:50, *v*/*v*). After a 5 min hold at 5% solvent B, elution was performed by a linear gradient from 5 to 55% solvent B in 55 min, from 55 to 95% solvent B in 10 min, followed by 10 min of maintenance, from 95 to 100% solvent B in 10 min, followed by 5 min of maintenance. The column effluent was split into two using a “T junction” placed after the chromatographic column and analyzed “on-line” both by UV-Vis and heated electrospray ionization mass spectrometry (HESI/MS); 80% of the effluent was sent to the Diode Array Detector (wavelength range 200–800 nm) while 20% of the effluent was analyzed by HESI/MS. Mass spectra were recorded from *m*/*z* 120 to 1800 in negative ionization mode. The capillary voltage was set at −27 V, the spray voltage was at 4 kV, and the tube lens offset was at −87 V in the first case while, in positive ion mode, the capillary voltage was set at 4 V, the spray voltage was at 4 kV, and the tube lens offset was at 140 V. The heater temperature was set at 200 °C and the capillary temperature was 275 °C. Data were acquired in MS and MS^n^ scanning mode.

#### 3.5.5. Quantification by RP–HPLC–DAD

To quantify the phenolic compounds, the HPLC system Agilent 1290 Infinity II LC, equipped with a quaternary pump and a Diode Array Detector (Agilent Technologies Italia SpA, Cernusco sul Naviglio, Milano, Italy) was used. Samples to analyze were dissolved in deionized water at 1 mg/mL concentration and filtered through a Chromafil syringe filter, pore-size 0.45 μm (Macherey-Nagel GmbH & Co., Duren, Germany). A measurement of 20 µL was loaded onto the column as described in 3.5.4 and the compounds were eluted by the same method reported above. Quantification of GA, PCA, and PHBA was carried out using calibration curves generated from serial dilutions of the corresponding analytical standards. The calibration equations and coefficients of determination (R^2^) were as follows:

GA: y = 3144.7x, R^2^ = 0.98 (2.5–7.5 μg)

PCA: y = 1489.6x, R^2^ = 0.99 (2.5–7.5 μg)

PHBA: y = 2598x, R^2^ = 0.98 (1.0–5.0 μg)

Quantitative results were expressed as μg of phenolic compound/mg fraction.

### 3.6. Cell Cultures

Human primary fibroblasts, apparently healthy (8400) and FRDA 3816, which has moderate homozygous (GAA) repeat expansions of 323 and 490 in the first introns of the *FXN* gene, were obtained from Coriell Repositories (Camden, NJ, USA). Cells 4497—(GAA) repeat expansions of 526 and 826—were obtained from the FRDA Cell Line Repository at the University of Texas Southwestern Medical Center (Dr. M. Napierala). Cells were grown in DMEM/F12 supplemented with 15% Fetal Bovine Serum, 1% Penicillin, Streptomycin, and 1X MEM Non-essential amino acids. DPBS, trypsin-EDTA, media, and supplements were from Thermo Fisher (Waltham, MA, USA); FBS was from Sigma-Aldrich (St. Louis, MO, USA). Cells were grown at 37 °C in a 5% CO_2_ humidified atmosphere, and they were used between passages 14 and 22.

### 3.7. Erastin/RSL3 Assay

The assay was conducted as described in Cotticelli et al. [30] with minor modifications. Briefly, FRDA fibroblasts were seeded in 48-well plates at a density of 8000 cells/well. The following day, medium was changed and erastin at the indicated concentration was added in fresh medium containing 1% FBS. After 2 h, a compound or its carrier control (water) was added to the wells. Viability measurements were performed the following day by using the Cell Titer-Glow Luminescent Cell Viability Assay (#G7571) from Promega (Madison, WI, USA) as per the manufacturer’s instructions. A fraction of lysates was then transferred into opaque white-wall plates and chemiluminescence readings were performed on a Synergy H1 microplate reader (Biotek, Winooski, VT, USA). Similarly, in the RSL3 assay, cells were seeded at a density of 5000–8000 cells/well in 96-well, black-wall, clear-bottom plates. The following day, medium was changed and RSL3 at the indicated concentration, was added in fresh medium containing 1% FBS and, after 2 h, a compound or its carrier control (water) was added to the wells. After 24 h, viability was measured as described above. When compounds in DMSO were added to the cultures, the DMSO was always kept below 0.5% (*v*/*v*) to avoid DMSO-related toxicity, generally detectable at 2% (*v*/*v*). An equal volume of DMSO was added to cells that were not treated with drugs (NT). When compounds in water were added to the cultures, an equal volume of water (0.5% *v*/*v*—carrier control) was added to cells where the compounds were not added. We did not detect any interference between any extract or compound we used and the Cell-Titer Glow.

### 3.8. Lipid Peroxidation Assay

FRDA fibroblasts were seeded at a density of 5000 cells/well in 96-well, black-wall, clear-bottom plates. The following day, medium was changed and RSL3 at the indicated concentration was added in fresh medium containing 1% FBS. After 24 h, medium was changed and Bodipy™ 581/591 C11 was added at a final concentration of 10 µM. After 30 min of incubation at 37 °C, cells were washed three times with DPBS, and fluorescence was measured at 488 _excitation_/510 _emission_ and 581 _excitation_/591 _emission_ wavelengths. The fluorescence ratio emission at 520 nm [oxidated]/591 nm [reduced] was calculated for the cells that were not treated with RSL3 and set to 1.

### 3.9. Frataxin Knock-Down Assay

Apparently healthy human fibroblasts (Coriell 8400) were seeded in 60 mm dishes at 20,000 cells/dish. On day 1, cells were transfected overnight using 10 nM double-strand small interfering RNA (siRNA) against frataxin mRNA (#SR301654A Origene, Rockville, MD, USA) or a control siRNA (5′ CAGUUUGCCCGGGAACCCACGGCGUGA-3′). The following day (day 2) medium was exchanged and PCA was added. The procedure was repeated on day 4 and day 5 and the cells were counted on day 6 using the Countess^®^ Cell Counter (Thermo Fisher, Waltham, MA, USA). Double strand siRNAs were transfected using RNAimax reagent (13778150 Thermo Fisher, Waltham, MA, USA) according to the manufacturer’s instructions. Frataxin protein was measured using the human Frataxin ELISA kit (ABCAM, Waltham, MA, USA).

### 3.10. Real-Time PCR

FRDA cells were seeded in 60 mm dishes at 300,000 cell/dish. The following day, PCA, OMAV, or carrier control was added. RNA was extracted 24 h later using Trizol^®^ (Thermo Fisher, Waltham, MA, USA); cDNA was prepared using the QuantiTect reverse Transcription kit (Qiagen, Germany), and RT-PCR was run using an Applied Biosystems ViiA 7 Real-Time PCR System (Thermo Fisher, Waltham, MA, USA Thermo Fisher) and QuantStudio software. Relative quantification was performed using the ∆∆CT method using TATA binding protein (TBP) as reference gene.

Taqman primers /probes (Thermo-Fisher, Waltham, MA, USA):

Hs00167524_m1 *ALOX 12*

Hs00989766_g1 *GPX4*

Hs009775964_g1 *NFE2L2*

Hs00920494_m1 *TBP*

### 3.11. Statistical Analysis

The *p*-values were derived from Student’s *t* test and are two-sided. Otherwise, *p*-values were calculated by one-way or two-way ANOVA with *post hoc* Bonferroni correction for multiple comparisons (Graph Pad Prism v8.1). A *p*-value < 0.05 was considered significant. We generally repeated the experiments in their entirety to confirm results. Because primary cells change with passage numbers, we generally do not combine data from independent experiments.

## 4. Conclusions

We studied the possible uses of chestnut shells, which are nowadays an under-exploited waste produced in huge amounts by the industrial chestnut peeling process. We obtained an aqueous extract, prepared by an eco-friendly and easily scalable technique, which we tested for anti-ferroptotic activity in Friedreich Ataxia, a neuro- and cardio-degenerative disease with very limited treatment options. We followed the biological activity through the molecular weight fractionation of the whole extract and the identification of PCA, GA, and PHBA as the major components of the polyphenolic extract. In doing so, we revealed the anti-ferroptotic activity of a high molecular weight tannin fraction by an unknown mechanism, which we plan to study in the future. We also described the anti-ferroptotic activity of PHBA and highlighted PCA as a molecule whose mechanism of action might be beneficial in FRDA. A limitation of this study is that it relies on the use of primary FRDA patient fibroblasts, which are not an affected tissue, and that we have not yet expanded the study to other patient-derived lines. We plan to further validate PCA in the cellular models of affected cell types, such as cardiac and neuronal lines, and potentially in animal models. Our study demonstrates the great potential of some of the bioactive molecules contained in chestnut shells as anti-ferroptotic agents, which could ultimately be used in the treatment of FRDA, both alone or in combination with other active molecules.

## Figures and Tables

**Table 1:** Total phenolic and tannin contents in fractions A and D after fractionation of CSDE.

	CSDE (μg GAE/mg)	F A (μg GAE/mg)	F A (mg GAE in the Whole Fraction)	F D (μg GAE/mg)	F D (mg GAE in the Whole Fraction)
TPC	512 ± 7.8	444.47 ± 11.16	297.79 ± 8.94	173.28 ± 4.97	29.79 ± 2.06
TTC	338 ± 2.5	384.70 ± 7.38	257.75 ± 5.11		

CSDE: Chestnut Shells Dry Extract; TPC: Total Phenolic Content; TTC: Total Tannin Content; F: Fraction; GAE: Gallic Acid Equivalents. All determinations were performed in triplicate and results are expressed as mean ± SD.

**Table 2:** List of compounds identified in fraction D and fraction 7 by UHPLC-MS^n^. Retention time (t_r_), specific quasi-molecular ions, and fragment ions are reported for each compound.

Peak	t_r_ (min)	MS *m*/*z*	MS^2^ Ions *m*/*z*	Proposed Structure
**1**	**8.72**	**133 [M-H]^−^**	**115, 89, 87, 73**	**Malic acid**
**2**	**9.08**	**149 [M-H]^−^**	**131, 89, 59**	**Tartaric acid**
**3**	**9.47**	**353 [M-H]^−^**	**293, 191, 173, 155, 111**	**Caffeoyl-isocitric acid**
4	9.87	481 [M-H]^−^	301, 275	HHDP-glucose isomer
**5**	**12.44**	**191 [M-H]^−^**	**173, 111**	**Quinic acid**
6	13.16 *	331 [M-H]^−^	271, 169, 125	Galloylglucose isomer
7	13.19	481 [M-H]^−^	301, 275	HHDP-glucose isomer
8	17.95	331 [M-H]^−^	271, 169, 125	Galloylglucose isomer
9	19.59	169 [M-H]^−^	151, 125	Gallic acid
10	20.58	331 [M-H]^−^	271, 169, 125	Galloylglucose isomer
**11**	**26.45**	**483 [M-H]^−^**	**465, 331, 313, 169, 125**	**Digalloyl glucose isomer**
12	29.98	153 [M-H]^−^	109	Protocatechuic acid
13	33.85	443 [M-H]^−^	425, 399, 281, 237	Unknown
**14**	**36.44**	**483 [M-H]^−^**	**465, 423, 331, 313, 271, 211, 169**	**Digalloyl glucose isomer**
15	39.63	137 [M-H]^−^	93	*p*-Hydroxybenzoic acid
16	43.33	167 [M-H]^−^	152, 123	Vanillic acid
17	44.51	197 [M-H]^−^	183, 153	Syringic acid
18	84.53	517 [M-H]^−^	499, 455, 437	Bartogenic acid

Compounds indicated in bold were detected only in fraction D. * The retention time refers to the HPLC-UV chromatogram of fraction 7.

## Data Availability

The data presented in this study are available on request.

## Supplementary Materials

The following supporting information can be downloaded at https://www.mdpi.com/article/10.3390/molecules31010070/s1, Figure S1: Frataxin levels in knocked-down cells; Figure S2 ALOX12 expression level in FRDA 4497 cells.

## Abbreviations

The following abbreviations are used in this manuscript:

UHPLC-UV	Ultra-high performance liquid chromatography-ultraviolet
UHPLC-MS^n^	UHPLC-mass spectrometry
RP–HPLC–DAD	Reverse phase-HPLC-diode array detector
NFE2L2, NRF2	Nuclear factor Erythroid 2-related Factor 2
FRDA	Friedreich Ataxia
LOOH	Lipid hydroperoxides
ALOX	Arachidonate-lipoxygenase
GPX4	Glutathione peroxidase 4
OMAV	Omaveloxolone
CSDE	Chestnut shells dry extract
PHBA	*p*-hydroxybenzoic acid
PCA	Protocatechuic acid
BSO	L-buthionine (S,R)-sulfoximine
ARE	Antioxidant response element
GAE	Gallic acid equivalents
TPC	Total phenolic content
TTC	Total tannin content
CS	Chestnut shells
GE	Glucose Equivalents
GA	Gallic acid
F	Fraction
