# Supplementary material for: Polyphenol-Enriched Fraction from Chestnut Shells as a Source of Bioactive Compounds for Friedreich Ataxia
Source: Molecules. 2025 Dec 24;31(1):70. doi: 10.3390/molecules31010070 (PMC12786618; doi:10.3390/molecules31010070)
Supplement: Supplementary file 1 [file molecules-31-00070-s001.zip › molecules-3983289-supplementary.pptx]

## Slide 1
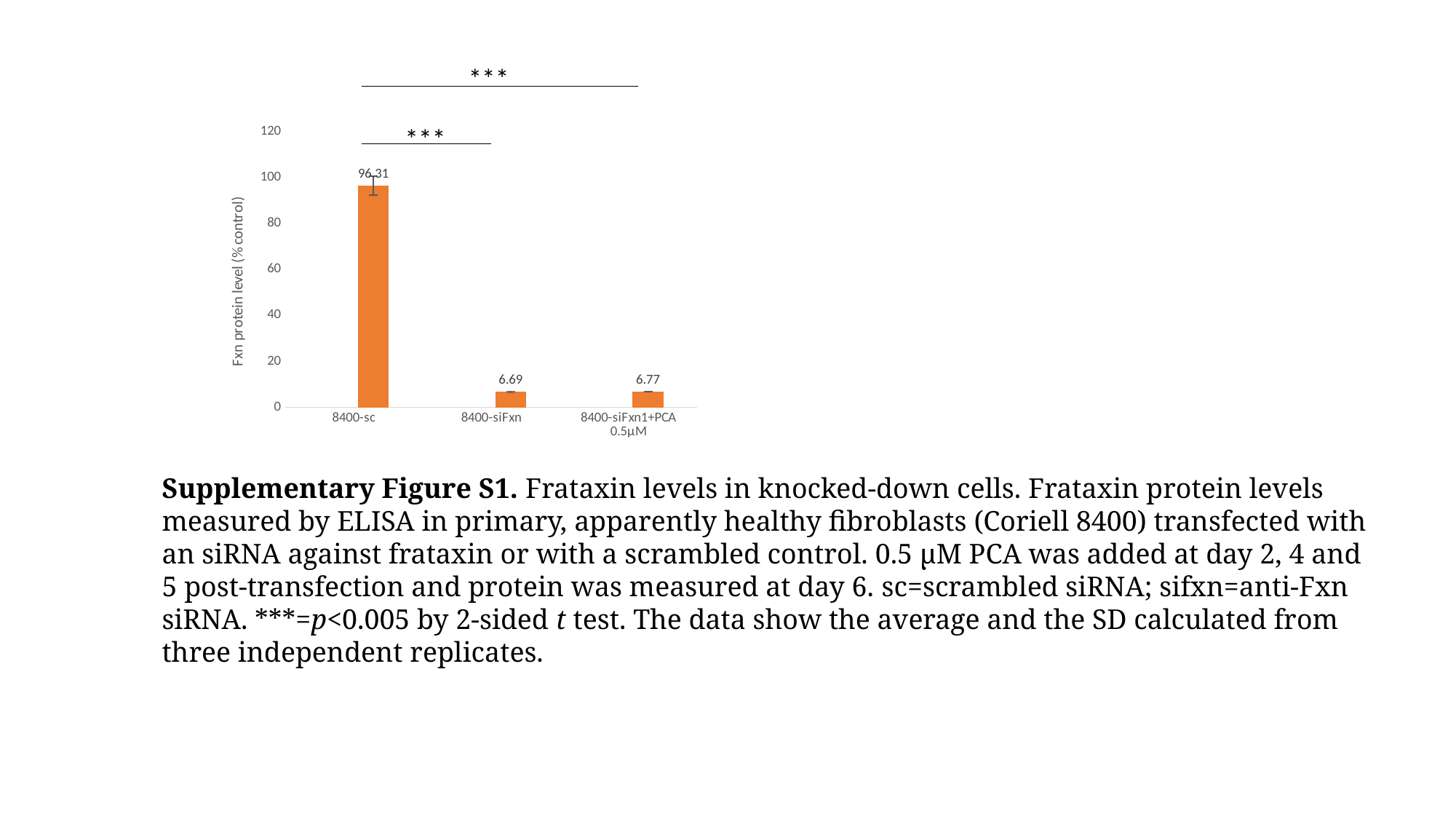

***
***
### Chart
| Category | | |
|---|---|---|
| 8400-sc | None | 96.30769230769229 |
| 8400-siFxn | None | 6.692307692307692 |
| 8400-siFxn1+PCA 0.5µM | None | 6.769230769230769 |Supplementary Figure S1. Frataxin levels in knocked-down cells. Frataxin protein levels measured by ELISA in primary, apparently healthy fibroblasts (Coriell 8400) transfected with an siRNA against frataxin or with a scrambled control. 0.5 µM PCA was added at day 2, 4 and 5 post-transfection and protein was measured at day 6. sc=scrambled siRNA; sifxn=anti-Fxn siRNA. ***=p<0.005 by 2-sided t test. The data show the average and the SD calculated from three independent replicates.

## Slide 2
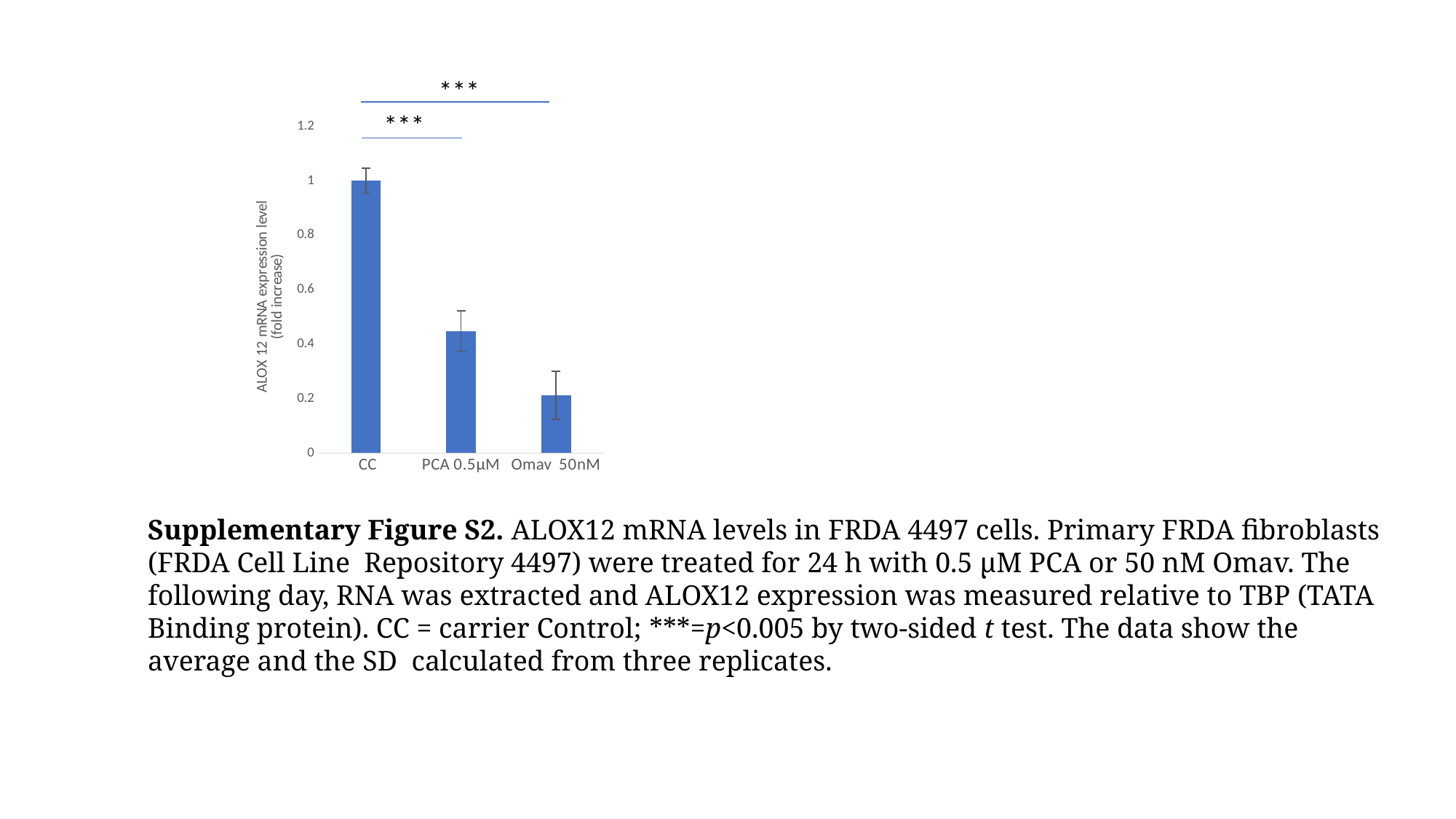

***
***
### Chart
| Category | |
|---|---|
| CC | 1.0007672816676039 |
| PCA 0.5µM | 0.448490262118533 |
| Omav 50nM | 0.212372329889565 |Supplementary Figure S2. ALOX12 mRNA levels in FRDA 4497 cells. Primary FRDA fibroblasts (FRDA Cell Line Repository 4497) were treated for 24 h with 0.5 µM PCA or 50 nM Omav. The following day, RNA was extracted and ALOX12 expression was measured relative to TBP (TATA Binding protein). CC = carrier Control; ***=p<0.005 by two-sided t test. The data show the average and the SD calculated from three replicates.
